# Blockade of the malignant phenotype by *β*-subunit selective noncovalent inhibition of immuno- and constitutive proteasomes

**DOI:** 10.18632/oncotarget.14428

**Published:** 2017-01-02

**Authors:** Bruno O. Villoutreix, Abdel-Majid Khatib, Yan Cheng, Maria A. Miteva, Xavier Maréchal, Joëlle Vidal, Michèle Reboud-Ravaux

**Affiliations:** ^1^ INSERM, U 973, Université Paris Diderot, Sorbonne Paris Cité, Paris, France; ^2^ INSERM, LAMC, U 1029, Pessac, France; ^3^ Sorbonne Universités, UPMC Université Paris 6, UMR 8256, ERL U1164, B2A, IBPS, Paris, France; ^4^ Institut des Sciences Chimiques de Rennes, Université de Rennes 1, UMR-CNRS 6226, Rennes, France

**Keywords:** proteasomes, immunoproteasome, noncovalent inhibitors, piperazine, virtual screening

## Abstract

A structure-based virtual screening of over 400,000 small molecules against the constitutive proteasome activity followed by in vitro assays led to the discovery of a family of proteasome inhibitors with a sulfonyl piperazine scaffold. Some members of this family of small non-peptidic inhibitors were found to act selectively on the *β*2 trypsin-like catalytic site with a preference for the immunoproteasome *β*2i over the constitutive proteasome *β*2c, while some act on the *β*5 site and post-acid site *β*1 of both, the immunoproteasome and the constitutive proteasome. Anti-proliferative and anti-invasive effects on tumor cells were investigated and observed for two compounds. We report novel chemical inhibitors able to interfere with the three types of active centers of both, the immuno- and constitutive proteasomes. Identifying and analyzing a novel scaffold with decorations able to shift the binders’ active site selectivity is essential to design a future generation of proteasome inhibitors able to distinguish the immunoproteasome from the constitutive proteasome.

## INTRODUCTION

**Figure 1:**
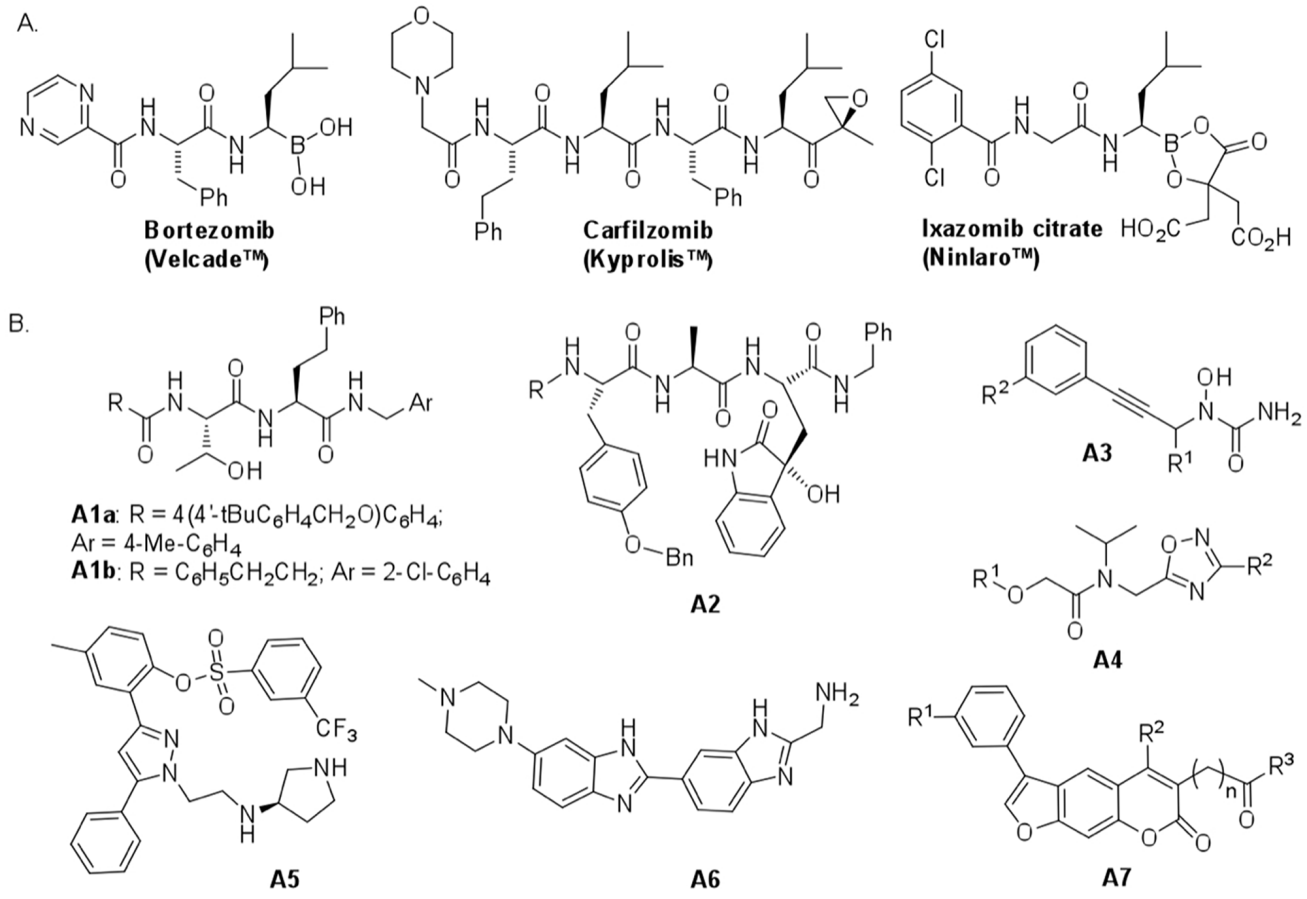
Chemical structures of some known proteasome inhibitors **A**. Inhibitors used in cancer therapy. **B**. Selected noncovalent inhibitors of proteasomes. Compounds A1a, A4, A5, A6 and A7 display noticeable activity for iCP.

The constitutive ubiquitin-proteasome system is mainly implicated in the controlled degradation of proteins in eukaryotic cells [1]. Since an increased degradation by proteasome of cell cycle inhibitors or proapoptotic proteins contributes to malignant transformation [2, 3], considerable efforts to develop proteasome inhibitors were made and led to three approved drugs, bortezomib for the treatment of multiple myeloma [4] and mantle lymphoma [5], carfilzomib [6] and the orally available ixazomib [7] for the treatment of multiple myeloma (Figure 1A). These covalent inhibitors inhibit mainly the *β*5 activity of the catalytic core of the constitutive proteasome (cCP) but also, indiscriminately, that of the catalytic core of the immunoproteasome (iCP). A large variety of other covalent inhibitors of the cCP have been reported [8, 9] whereas noncovalent ones are less frequent. Some molecules contain a *N*-capped dipeptide backbone (compounds A1a-b, Figure 1B) [10, 11] or mimic the natural cyclic tripeptide TMC-95 (compound A2) [12–14] (Figure 1B). Recently reported noncovalent inhibitors are now essentially organic compounds [15–21] (compounds A3-A7, Figure 1B). Since noncovalent inhibitors are in general devoid of reactive groups, they do not have the drawbacks generally associated with the presence of a warhead such as lack of specificity, instability, and excessive reactivity [9]. Moreover, the reversible binding provides enzyme-inhibitor complexes with a limited life-time and favors inhibitor widespread tissue distribution [10].

The immunoproteasome is found in lymphoid cells and cells exposed to cytokines like interferon-γ or tumor necrosis factor [22]. The lack of selectivity of bortezomib and carfilzomib against the cCP and iCP may in part explain some of the side effects and resistance observed during treatments with these molecules and that arise from undesired inhibition of the constitutive proteasome in normal cells [23, 24]. Expression of the immunoproteasome was found highly increased in a number of diseases [22] including cancer, autoimmune diseases and multiple myeloma [23] as well as rheumatoid arthritis [25], Huntington disease [26] and Alzheimer’s disease [27]. Taking together these observations, it would seem that targeting selectively the iCP could be critical in several disease indications [28].

The catalytic cores iCP and cCP are closely related with two outer rings of 7 α-subunits and two inner rings of *β*-subunits with subtle differences in the catalytic site subpockets [29]. The constitutive *β*-subunits *β*1, bearing the so-called caspase–like or post-acid activity (PA, often cleaves after acidic amino acids), *β*2, bearing the trypsin-like activity (T-L, often cleaves after basic amino acids) and *β*5 bearing the chymotrypsin-like activity (ChT-L, often cleaves after hydrophobic amino acids) are replaced in the iCP by three homologous subunits (*β*1i, LMP2; *β*2i, MECL; β5i, LMP7). Of importance, small compounds interfering with one type of catalytic activity can also block the others (cross-react) as observed for instance for the TMC-95 mimic A2 (Figure 1B) [12–14] that binds to all three proteosomal active centers. Some compounds can cross-react between iCP and cCP and also between proteasomes of different species [8, 9, 29, 30]. Furthermore, catalytic activities most often, depending on the chemistry and the nature of the ligand, require the presence of the adjacent subunit, with for instance, for the ChT-L activity, the catalytic site on the *β*5 subunit with additional contacts for ligand binding and orientation involving residues located on the *β*6 subunit. The enzymatic activity of each catalytic center is associated with the Thr1 *N*-terminal residue, in which the gamma hydroxyl group acts as a nucleophile in the hydrolysis of the peptide bonds.

Only a small number of iCP-selective inhibitors have been described [8, 31, 32]. They are mainly covalent inhibitors bearing an electrophilic warhead (peptide epoxyketone [33–35], peptide aldehyde [23], peptide sulfonyl fluoride [36], peptide chloroacetamide [37], oxathiazolone [38] or *β*-lactone [39]). Conversely, noncovalent inhibitors of the immunoproteasome are still poorly described. Some *N*-capped dipeptides (e.g., molecule A1a) are selective inhibitors of the ChT-L activity of iCP [10, 40] (Figure 1B). Pyrazole compound A5 inhibits the ChT-L and PA activities of both iCP and cCP [19]. The oxadiazole scaffold of compound A4 was discovered by structure-based virtual screening and led to the development of potent inhibitors of the ChT-L activity of the cCP [17] that are less active against iCP (unpublished result; IC_50_ = 0.14 ± 0.02 μM instead of 0.037 ± 0.003 μM for cCP, R^1^ = 4-BrC_6_H_4_, R^2^ = 3,4-di-MeOC_6_H_3_). Benzimidazole derivative A6 acted selectively on the T-L activity of iCP (subunit *β*2i) [20]. Psoralene based inhibitors (e.g., compound A7), selective of the ChT-L activity of iCP, were also recently reported [21].

Overall, it would be highly desirable to identify novel immunoproteasome-selective inhibitors with new scaffolds. For the reasons commented above, noncovalent iCP inhibitors may constitute a valuable alternative to covalent ones. For example, the knowledge of cCP binders can be exploited to find iCP inhibitors as observed with the *β*1/*β*2 specific sulfonamide derivative that noncovalently binds between subunits *β*1 and *β*2 [41].

In this study, we investigate small organic inhibitors that were initially identified using hierarchical structure-based virtual ligand screening computations [42] performed in the ligand-binding subsites present on the *β*5/*β*6 subunits of the cCP [15]. Displaying a new piperazinyl sulfonamide scaffold (Table 1), some inhibitors act selectively on the *β*2c and *β*2i subunits (with a preference for the *β*2i) or, simultaneously on the *β*5 and *β*1 subunits of both the iCP and cCP. Their differential inhibitory efficiency on the three types of proteasomal activities was evaluated *in vitro* together with their cytotoxicity, anti-proliferative and anti-invasive effects on two cancer cell lines.

**Table 1:** Inhibition profile of human iCP (in bold) and cCP (plain text) at pH 8.0 and 37°C by piperazinyl sulfonamides^a^

Compd	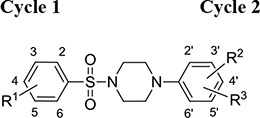			IC50 (μM)b, % inhibition or x (activation factor) at 50 μM		
	R1	R2	R3	ChT-L	PA	T-L
**B1a**	4-CH_2_CH(CH_3_)_2_	4’-NO_2_	H	**ni** ni	***25 %*** ni	**7.6 ± 0,6** 10.3 ± 0.5
**B1b**	4-C(CH_3_)_3_	2’-CH_3_	3′-Cl	**ni** ni	**ni** ni	**2.6 ± 0.4** 12.0 ± 1.1
**B1c**	4-C(CH_3_)_3_	2’-NO_2_	4’-Cl	**ni** ni	**ni** ni	**3.3 ± 0.4** 8.1 ± 0.8
**B1d**	4-Cl	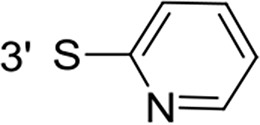	4’-NO_2_	**ni**	***46 %***	**x 4.7**
**B1e**	4-CH_3_	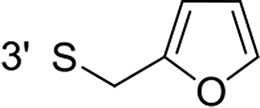	4’-NO_2_	**5.5 ± 0.3** 2.5 ± 0.1	**3.3 ± 0.3** 4.2 ± 0.1	**x 3** ni
**B1f**	4-Cl	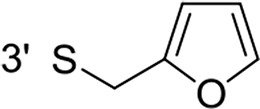	4’-NO_2_	**5.8 ± 0.2** 3.2 ± 0.1	**3.7 ± 0.2** 7.4 ± 0.3	**x 3** ni
**B1g**	4-C(CH_3_)_3_	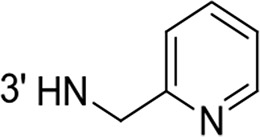	4’-NO_2_	**24.6 ± 3.5**	**13.9 ± 1.4**	**ni**
**B1h**	4-NHCOCH_3_	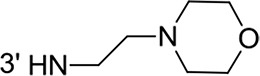	4’-NO_2_	***50.5 %***	***45 %***	**ni**
**B1i**	3,4-OCH_3_	3′-NHCH_2_C(CH_3_)_3_	4’-NO_2_	**ni**	**ni**	**ni**
	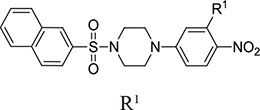					
**B2a**	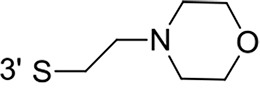			**32.4± 0,7**	**31.2 ± 1.0**	**ni**
**B2b**	H			***58.9 %***	***50 %***	**x 4.5**
**B3**	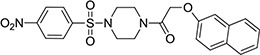			**6.7 ± 0.2** 6.5 ± 0.3	**7.1 ± 0.2** 8.1 ± 0.6	**ni** ni
**B4**	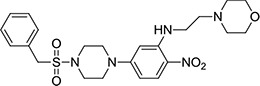			**ni**	**ni**	**ni**

^a^The inhibition was evaluated after 15 min incubation of the enzyme with the compounds before adding the appropriate fluorogenic substrate to evaluate the remaining ChT-L, PA and T-L activities. ^b^The IC_50_ values were calculated by fitting the experimental data to eq 1 or eq 2. ni: inhibition < 25% at 50 μM.

## RESULTS AND DISCUSSION

### Compounds B1a-c target the 20S proteasome β2 subunits with a preference for the immunoproteasome whereas the other compounds target simultaneously the β1 and β5 subunits

**Figure 2:**
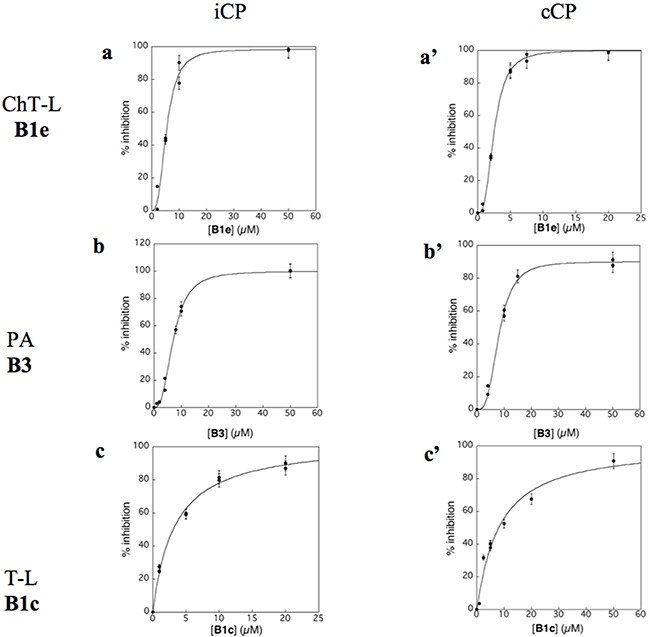
Inhibition profile of human iCP (a,b,c) and cCP (a’, b’, c’) by compounds B1e (a,a’), B3 (b,b’) and B1c (c,c’) at pH 8.0 and 37°C [iCP] = [cCP] = 0.3 nM; ChT-L activity, [Suc-LLVY-AMC]_0_ = 20 μM; PA activity, [Z-LLE-βNA]_0_ = 100 μM (iCP) and 50 μM (cCP); T-L activity, [Boc-LRR-AMC]_0_ = 50 μM. The experimental data are adjusted to equation 1 (c) or equation 2 (a, a’, b, b’, c’).

Virtual screening was carried out on the mammalian cCP proteasome using several computational approaches and a short consensus list of molecules were selected, purchased from the ChemBridge corporation and evaluated experimentally. These hits are here further characterized using several complementary approaches. The *in silico* screening efforts [15] led to the discovery of a family of piperazine sulfonamide binders inhibiting at least one type of activity of cCP. Among the 65 hits that we identified, a dozen of molecules belonged to this category (Table 1). We decided to explore further their properties by testing their potential to inhibit human iCP *versus* cCP. They were assayed for *in vitro* inhibition of purified human cCP and iCP by measuring the hydrolysis of the *β*5 (ChT-L) substrate Suc-LLVY-AMC, *β*1 (PA) substrate Z-LLE-βNA, and *β*2 (T-L) substrate Boc-LRR-AMC in the presence of various concentrations of the tested molecules. When a noticeable inhibition was detected, the IC_50_ values were determined (Figure 2). Structures and inhibition efficacies of the most efficient molecules are summarized in Table 1.

From a structural point of view, the compound sulfonyl group is linked to the piperazinyl moiety and to cycle 1, a diversely substituted cycle by phenyl (B1a-i, B3), naphthyl (B2a-b) or benzyl (B4) groups. The second piperazinyl nitrogen is linked to monosubstituted (B1a, B2b) or disubstituted (B1b-i, B2a, B4) phenyl cycle 2. For B3, the spacer CO-CH_2_-O ensures the covalent link between piperazinyl nitrogen and a naphthyl group. Compounds B1a-c inhibited selectively the T-L activity of both, the iCP and cCP. A poor influence of the nature of cycle 1 R^1^ substituent, [isobutyl (B1a) or *tert*-butyl (B1b-c)] and cycle 2 ones [R^2^ = 4’-NO_2_ (B1a), R^2^ = 2’-CH_3_ and R^4^ = 3′-Cl (B1b); R^2^ = 2’-NO_2_ and R^4^ = 4’-Cl (B1b)] was observed for cCP with IC_50_ values ranking from 8.1 ± 0.5 to 10.3 ± 0.8 μM. Compounds B1a, B1b and B1c were more efficient on iCP than on cCP with cCP/iCP ratios for the IC_50_ values of 1.4, 4.6 and 2.4, respectively. The introduction of pyridyl (B1d and B1g), furanyl (B1e and B1f) or morpholino (B2a and B1h) heterocycles in the 3′-cycle 2 substituent failed to maintain the T-L inhibitory activity whereas ChT-L and PA activities were both inhibited. This indicates that cycle 2 3′-substituents were not tolerated in the β2 subunit binding region. The PA activity of iCP was slightly more efficiently inhibited by B1e and B1g than the ChT-L one (factor of ≈ 2.2) whereas the opposite was observed for the inhibition of cCP (factors of 1.7 and 2.3 for B1e and B1f respectively). The ratio cCP/iCP was respectively of 1.3 and 2 for B1e and B1f (PA activity), and 0.55 and 0.45 (ChT-L activity). For both enzymes, the attachment of the heterocyclic substituent at the 3′ position of cycle 2 seemed more favorable using the arm S(or NH)CH_2_ (B1e-g) than a shorter (B1d) or a longer (B1h) one. Changing cycle 1 R^1^, 4-CH_3_ (B1e) to 4-Cl (B1f), retained the *in vitro* ChT-L and PA inhibitory activities. The replacement of the phenyl cycle 1 by the bicyclic naphthyl (B2a) was detrimental, mostly if the 3-substituent (NH-CH_2_-furane) was replaced by hydrogen. Conversely, the replacement of cycle 2 by the motif COCH_2_O-naphthyl (B3) led to a molecule inhibiting quasi-equally the ChT-L and PA activities of both iCP and cCP. These results highlight the importance of the nature of the substituent present on cycle 2 of the piperazine sulfonylamide central core for either the selective inhibition of the T-L activity, or for the simultaneous inhibition of the ChT-L and PA activities. Moreover, these substitutions may be used to guide preferentially the inhibition towards iCP *versus* cCP. A selective inhibition of the proteasomal T-L activity was also observed with a related quinoline-sulfonyl hybrid [43].

### The subunit preference is supported by computational analysis of selected compounds

Crystal structures of murine iCP and cCP complexes provided valuable insights over the subtle and complex structural differences among the different active sites [29]. For instance, subunit *β*5i can accommodate larger amino acid side chains at position P1 than *β*5c. This difference in size was partly attributed to distinct conformation of Met 45. The opposite is expected for the P3 residue due to Ser 27, which is substituted by Ala in *β*5c. The S3 pocket is more hydrophilic in *β*5i as compared to *β*5c. Differences were also noticed for the S1 pocket of *β*1i, which is more hydrophobic and smaller than that of *β*1c, and for the subunit *β*1i where the S3 pocket is of smaller size and more polar than that of *β*1c. On the other hand, all *β*2 subunits harbor a relatively large S1 pocket and overall *β*2c and *β*2i are structurally very similar in the substrate-binding channel.

The exact position of the here identified inhibitors in the different catalytic centers would require X-ray crystallography studies but attempts to obtain crystals for the presented compounds have so far failed. Yet, it has been possible to crystallize some sulfonamide ligands at the *β*1*/β*2 interface [41] related to the compounds that we identified by virtual screening [15], indicating that molecules selected by computational means can help gaining knowledge over this highly complex enzymatic system. To facilitate the reading of the structural and chemical data, we decided to present the analysis of two inhibitors that are chemically related but possess different properties and to rationalize their likely mechanism of actions in the light of the recently reported human cCP crystal structure [44] and of several other structural studies [11, 29, 45, 46]. The B1b and B1e inhibitors were docked with two different methods in all three binding centers of the constitutive human proteasome and compared with the mouse immuno- and constitutive proteasomes.

**Figure 3:**
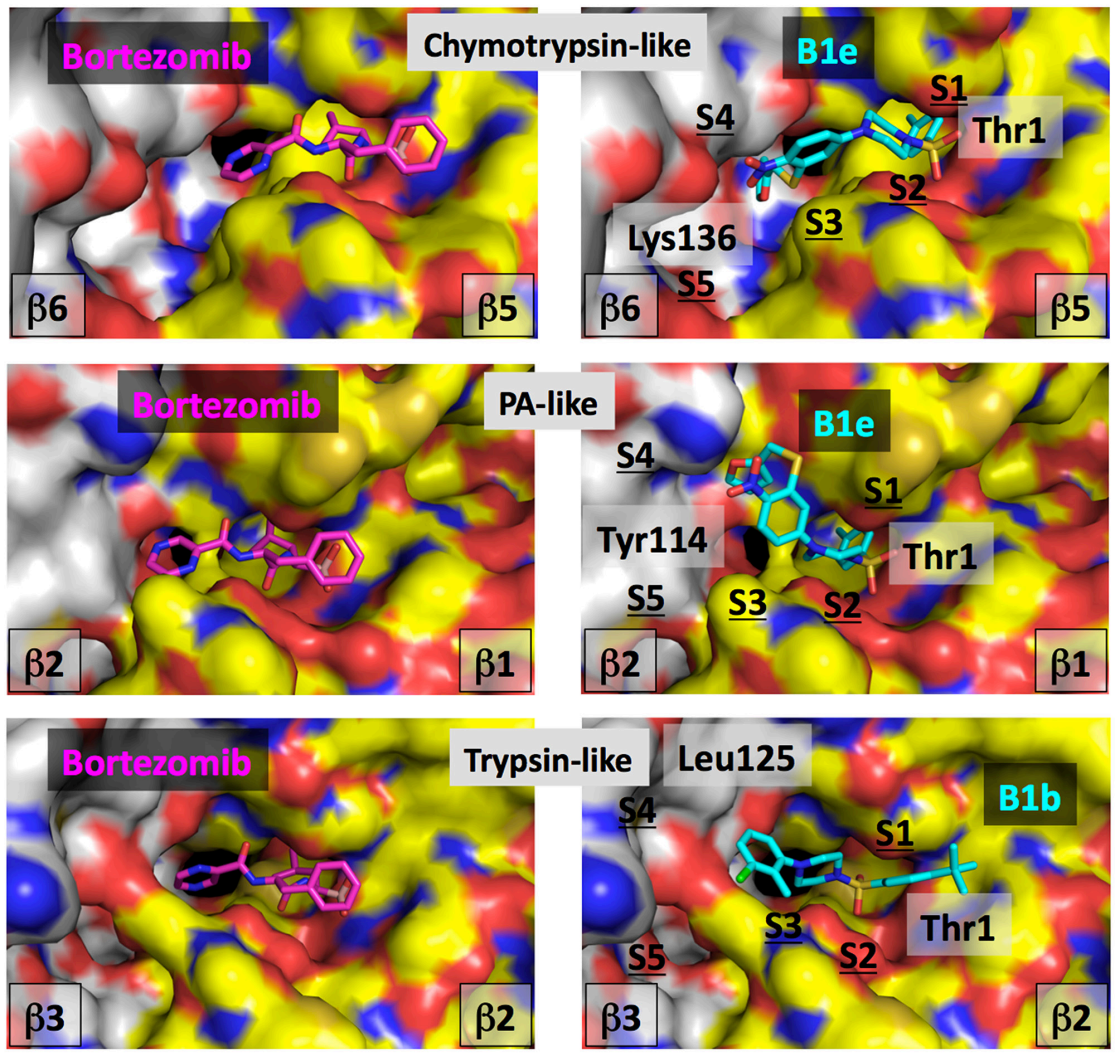
Computational analysis of compounds B1e and B1b Left, bortezomib is shown co-crystallized with the human cCP in the catalytic centers. Right, compounds B1e or B1b are positioned into the equivalent centers by computational approaches. The same color-coding is applied in all the images. The molecular surface of the catalytic subunit is shown in yellow (C atoms), blue (N atoms), red (O atoms) and brown (S atoms) while for the surface of the adjacent subunit, the color is white (C atoms), blue (N atoms), red (O atoms) and brown (S atoms). The color code for the bortezomib atoms is: C atoms are in magenta, N atoms in blue, O atoms in red and the B atom is in light red. For compounds B1e and B1b, C atoms are in cyan, O are in red, N are in blue and S are in yellow. Some residues are labeled for orientation (see text).

The most likely position of inhibitor B1e in the ChT-L binding site of cCP is shown in Figure 3. In this orientation the sulfonyl group would make hydrogen bonds with Thr 1 and Gly 47, two important residues of the active site known from X-ray crystallography studies to interact with such chemical group in a similar fashion [41, 46, 47]. In this orientation, the 4-CH_3_-phenyl group of molecule B1e could nicely fit into the hydrophobic and relatively large S1-specificity pocket and have favorable interactions with Met 45, an important amino acid of this subpocket. This orientation would thus mimic the P1-leucine side chain of the covalent bortezomib inhibitor co-crystallized with the human proteasome molecule or of the *C*-terminal benzylic group of compound A1b [11] and A2 [12] also co-crystallized with the proteasome. The piperazine group of B1e could have additional hydrophobic interactions and form several hydrogen bonds in the S2-S3 subsites. The nitrogen atoms would indeed adopt a position relatively similar to the ones observed for bortezomib. Other hydrophobic contacts and polar contacts are seen in the large S4 and S5 subsites and the NO_2_ group of B1e is here expected to form hydrogen bonds with the protein backbone and possibly with the Lys 136 side chain of the *β*6 subunit. Such interactions of a NO_2_ group would be consistent with the type of interactions that such chemical function would make. In fact we analyzed over 50 X-ray structures downloaded from the Protein Data Bank that contain a NO_2_ group including the nitro-biaryl ether macrocycle derived from TMC-95 in complex with the proteasome [45]. Nitro groups NO_2_ tend to have interactions with backbone atoms, but interact very often with the Lys and Arg side chains and can also have favorable interactions with Asn and His. All these contacts could take place in the iCP as suggested by the structural alignment of mouse iCP and cCP over the structure of human cCP. Interestingly, none of the 20 generated poses allowed similar types of positioning and interactions for the inhibitor B1b that was found inactive on the ChT-L site. In fact, even if the sulfonyl group were to be oriented differently, the hydrophobic regions of the B1b molecule would tend to point toward polar or charged amino acid side chains. Thus, B1b, with its two hydrophobic heads, does not seem to fit properly in the chymotrypsin-like sites.

With regard to the PA site, it can be seen in Figure 3 that compound B1e could fit in this active center in a tilted position as compared to the one observed in the ChT-L center. In the *β*1 subunit, the S1 specificity pocket contains for instance Arg 45 but the hydrophobic head of the B1e inhibitor can make some favorable contacts with the hydrophobic moiety of the Arg residue, a situation in part observed in the co-crystal structure of the bortezomib-human constitutive proteasome complex. The somewhat titled orientation of B1e as compared to bortezomib (Figure 3) in this catalytic center is in fact very similar to the tilt of inhibitor A1b co-crystallized with the proteasome ChT-L center [11]. The B1e inhibitor would make additional contacts with for instance Tyr 114 of the adjacent *β*2 subunit (an Asp in the equivalent position of the *β*6) and possibly hydrogen bonds can be formed between the NO_2_ group of the B1e compound and the nearby *β*2 His 116. Superimposition of the mouse cCP and iCP onto the human docked structure suggests that molecule B1e could interact equally well with both types of proteasome. Possibly, molecule B1b, that does not inhibit significantly the *β*1 site, is too small to make sufficient hydrophobic contacts with residues of the *β*2 subunit in the S4-S5 subpocket area while hydrogen bonds cannot form in the S4 region.

In the T-L center (*β*2), molecule B1e was found to be essentially inactive while molecule B1b was active. In Figure 3, a possible position of B1b is seen. The molecule occupies the site in a similar fashion than bortezomib except for the S1 site. The T-L S1 pocket is large and it was found that the P1-leucine residue of bortezomib does not form hydrophobic contacts there [48]. In our docking study, it is not clear if the hydrophobic and aromatic cycle 1 of inhibitor B1b points in the S2 pocket, like the phenylalanine P2 residue of bortezomib or if this cycle points into the S1 pocket. Considering the size of the S1 pocket, this could be definitively possible without changing the overall orientation of the compound although this may not be favorable from an energetic point of view as this pocket contains in the human cCP the negatively charged Asp 53 residue, also present in the mouse enzyme while it is Glu 53 in the mouse immunoproteasome. The S1 pocket is thus essentially hydrophilic and in part negatively charged and this might be the reason why no docking poses could fit the molecule such that it makes contact with the S1 subpocket and still has a relatively good predicted binding score. In this orientation, hydrogen bonds could form between the B1b molecule and sub-pockets S2 and S3.

The other hydrophobic ring of inhibitor B1b could have hydrophobic interactions in the area of the S4 subpocket, for example with residues Leu 125 and Ile 126, a situation highly related to the interaction found for a phenyl group distal to the P1 of the larger LU-102 inhibitor (azido-Phe-Leu-Leu-4-aminomethyl-Phe_(P1 residue)_-methyl vinyl sulfone) co-crystallized with yeast proteasome (LU-102 is interacting with the equivalent Leu and Ile residues in yeast) [46]. Nearby Leu 125, three negatively charged residues are found in the human cCP, *β*2 Glu 22 and *β*3 Asp 124 and Glu 105 that should repel the NO_2_ group of B1e. This situation is conserved in the mouse iCP although Glu 22 is an Asn but at position 23, an Asp is found (Gly in the human cCP). Taken together, the presence of a negative charge in the S1 pocket and in the S3-S4-S5 areas would not favor the binding of molecule B1e due to unfavorable contacts with cycle 1 and the NO_2_ group of cycle 2 while it could be possible to fit the more hydrophobic B1b molecule.

### Inhibition of tumor cells proliferation and invasion by B1f and B3 compounds

**Figure 4:**
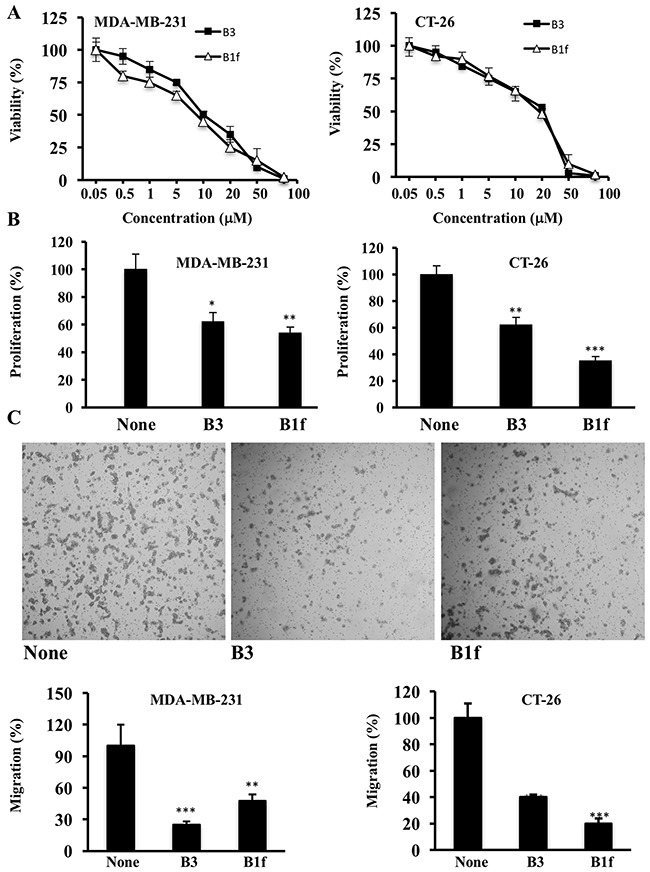
Effect of compounds B1f and B3 on tumor cell proliferation and invasion **A**. CT-26 and MDA-MB 231 cells were treated with various concentrations of indicated compounds for 48h and cells viability was measured by MTT assay. **B**. Tumor cells were serum deprived overnight and then treated for 24 h with control media (None) or media containing compound B3 or B1f. Cell proliferation was assessed using Cell Titer96 non-radioactive cell proliferation assay. Results are shown as means ± S.E. of three experiments performed in triplicate. *P < 0.05. **P < 0.001. *** P < 0.0001. **C**. Indicated tumor cells were incubated in a microchemotaxis chamber pre-coated with collagen IV for cell invasion assay as described in the Materials and Methods section. The results are represented as the percentage of invading cells. Data are representative of 3 experiments and shown as mean ± S.E. (n=3 per group). **P < 0.001. *** P < 0.0001.

We first analyzed the cytotoxic effects of B1f and B3 treatment on colon (CT-26) and breast cancer cells (MDA-MB-231 cells) using the MTT cell viability assay. B1f and B3 inhibited the viability of these cells in a dose-dependent manner after 48 h treatment. Higher doses of B1f and B3 lead to 100% cytotoxicity (Figure 4A). For MDA-MB-231 cells the LC50 values were 15.4 ± 4.7 and 45.8 ± 5.2 μM for B3 and B1f, respectively. For the CT26 cells the LC50 values were 26.4 ± 1.7 and 24.4 ± 8.6 μM for B3 and B1f, respectively. We next assessed the effect of B1f and B3 on the proliferation of these cells during 24h. As can be seen, in the presence of 10 μM of B1f and B3 compounds, tumor cells exhibited significant reduction in their proliferation in comparison to control cells (Figure 4B). Similarly, to evaluate whether these inhibitors can affect their invasion, tumor cells were incubated for 24 h in a microchemotaxis chamber pre-coated with collagen IV (Figure 4C) in the presence of the compounds B1f or B3. Here again, treatment of these cells with the inhibitors (10 μM) clearly decreased their ability to invade (Figure 4C).

**Figure 5:**
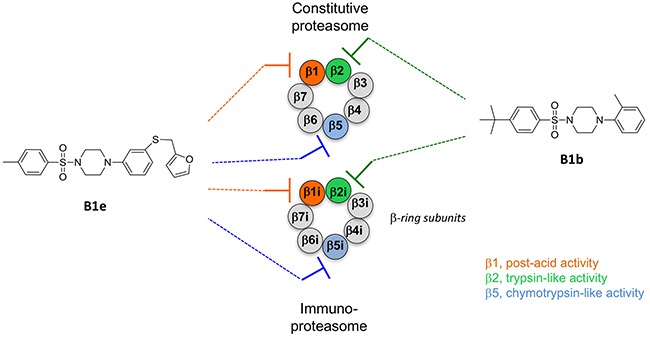
Schematic representation of the β-ring subunits of the cCP and iCP and their inhibition by representative sulfonyl piperazines

The discovery of piperazine sulfonamides broadens the range of potential scaffolds able to inhibit the immunoproteasome. Moreover, compounds that inhibit the trypsin activities of iCP and cCP as summarized in Figure 5 are still relatively rare. Several here described compounds constitute interesting starting points for the development of noncovalent immunoproteasome-selective inhibitors susceptible to target selectively the malignant phenotype of cancer cells.

## MATERIALS AND METHODS

The compounds selected by virtual ligand screening were purchased from ChemBridge corporation (www.chembridge.com). Purified human constitutive 20S proteasome cCP and human 20S immunoproteasome iCP from erythrocyte were obtained from Boston Biochem (Cambridge, USA). All fluorogenic substrates (Suc-LLVY-AMC, Boc-LRR-AMC, and Z-LLE-*β*NA) were obtained from Bachem (Weil am Rhein, Germany). Other reagents and solvents were purchased from commercial sources. Fluorescence was measured using a BMG Fluostar microplate reader (black 96-well microplates).

### Computational analysis

The virtual screening procedure initially used to identify the inhibitors was previously described [15]. Briefly, the *in silico* screening procedure was performed on the cCP ChT-L active site and using the 3D structure of the bovine proteasome [49] (the human and bovine subunits that carry the catalytic activity are 94-100% identical). The 3D structure used (PDB code 1IRU) was prepared for virtual screening computations (e.g., addition of hydrogen atoms at pH 7, short energy minimization, etc). Several docking software packages were used including Fred (OpenEye Scientific Software, Santa Fe, NM), Surflex [50] and LigandFit [51] to screen the ChemBridge compound collection (about 400,000 compounds that were filtered with our tool FAF-Drugs to remove compound with unwanted physicochemical properties [52]). The filtering process led to a collection containing about 300,000 molecules (about 180,000 clusters with families containing a few members up to about 20-30 members) that were then docked into the catalytic site. A consensus list of molecules possessing the best predicted binding scores with Surflex and LigandFit was generated and after visual inspection, the selected molecules were purchased from ChemBridge (www.chembridge.com) and tested experimentally. Prior to ordering the molecules, physicochemical properties, the search for structural alerts and for the presence of substructures potentially interfering with assays (e.g., PAINS) were investigated again with our online server FAF-Drugs3 [53]. With regard to physicochemical properties, the filtering was soft and, for instance, molecules with MW around 500 were tolerated as our goal was to probe these enzymes and find novel starting points. Similarly, the filtering on structural alerts was soft and we decided to keep aromatic NO2 as over 15 approved drugs contain such a group and because such group can be substituted during the optimization stages if needed. With regard to group potentially interfering with assays, we have noticed over the years that many such groups can be flagged after analysis of their structures but turn out to be inactive in various assays, to display structure-activity relationship (SAR), to co-crystallize with targets, to be present in approved drugs and indeed to be noninterfering and as such valuable chemical probes and starting points. We thus annotated our molecules but were permissive with this filtering step. For instance, compound B1a was flagged in silico as potential assay interfering compound but highly similar compounds are co-crystallized with targets and display SAR (e.g, PDB code 3CZR, target 11beta-HSD). The behavior of B1a (and other compounds) in our experiments support this selection process as if the molecules were to be interfering with the assays, they will be active/inactive on the different catalytic activities and small structural changes in the compounds would not impact affinity or selectivity. Selected ligands after experimental testing were prepared with our compound preparation package [53] and re-docked with the last version of Surflex and our online server MTiOpenScreen [54] into the three catalytic centers of the recently reported human cCP [44] taking into account several other structural studies [11, 29, 45, 46]. The twenty best-scored poses for each inhibitor were initially selected. The interactive structural analysis and figures were done with molecular graphics package PyMOL (Schrodinger LLC, USA). A good agreement for the lowest energy poses between the two docking packages was noticed and interestingly, even the docking performed over the entire surface of a pair of subunits via our server MTiOpenScreen led to the positioning of the compounds in the catalytic site with an orientation similar to the one observed by the two docking methods and with the lowest predicted binding scores. The selection of the final most likely pose for each molecule and for the molecules shown in this study was performed after analysis of the score, comparisons with experimental structures co-crystallized with ligands containing similar or related substructures/fragments and also after computations of various non-bonded interactions (e.g., salt-bridges, hydrogen bonds, pi-stacking…).

### Proteasome inhibition assays

Proteasome activities were determined by monitoring for 45 min at 37°C the hydrolysis of the appropriate fluorogenic substrate Suc-LLVY-AMC (ChT-L activity) and Boc-LRR-AMC (T-L activity) using λ_exc_ = 360 nm, λ_em_ = 460 nm, and Z-LLE-βNA (PA activity) using λ_exc_ = 340, λ_em_ = 405 nm, in the presence of untreated (control) or proteasome (iCP or cCP). Substrates and compounds were previously dissolved in DMSO. The buffers were (pH 8.0): 20 mM Tris, 10% (v/v) glycerol, 0.01% (w/v) SDS, and 2% (v/v) DMSO (ChT-L and PA activities); 20 mM Tris, 10% (v/v) glycerol, 0.01% (w/v). The final iPR and cPR concentrations were 0.3 nM using 20 μM Suc-LLVY-AMC (ChT-L), 50 μM Boc-LRR-AMC (T-L) and 100 μM Z-LLE-βNA (PA). Using the appropriate substrate, the compounds (0.1-100 μM) were tested in duplicate for each inhibitor concentration to detect their potential to inhibit the ChT-L, T-L, and PA activities. The enzyme and the inhibitors were incubated for 15 min before the measurement of the enzyme activity. Initial rates determined in control experiments (V_0_) were considered to be 100 % of the peptidase activity; initial rates (V_i_) that were above 100 % in the presence of a tested compound were considered to be activations (expressed as activation factor), while initial rates below 100 % were considered to be inhibitions. The inhibitory activity of compounds was expressed as IC_50_ (inhibitor concentrations giving 50 % inhibition). The values of IC_50_ were calculated by fitting the experimental data to the equation 1 or equation 2, where nH is the Hill number.

$$\%\text{Inhibition}=(100[I_{0}])/({\text{IC}}_{50}+[I_{0}])$$ (eq.1)

$$\%\text{Inhibition}=(100[{I_{0}}^{\text{nH}}])/({\text{IC}}_{50}+[{I_{0}]}^{\text{nH}})$$ (eq.2)

### Viability and proliferation assays

For the viability assay, CT-26 (colon cancer) and MDA-MB231 (breast cancer) cells were plated in triplicate on 96 wells plate (5.10^3^/well). After treatment with indicated drugs, cells were incubated for 48 hours at 37°C and incubated with solution of 5 mg/ml of 3-(4,5-dimethylthiazol-2-yl)-2,5-diphenyltetrazolium bromide (MTT) tetrazolium substrate (Sigma) for 30 minutes at 37°C prior analysis on a microplate reader at 570. The LC50 value was defined as the drug concentration causing 50% cell death (LD = lethal dose) compared to growth of the untreated control cells. For cell proliferation, cells were plated in triplicate on 96 wells plate (5.10^3^/well) under serum free conditions for 24 h. The starved cells were then incubated for 24h with fresh media containing the two selected compounds B1f and B3 that inhibit both ChT-L and PA activities of iCP and cCP. These two compounds were chosen based on reported studies describing that the simultaneous inhibition of ChT-L and PA activities by two selective inhibitors of these activities seemed to enhance the anticancer efficiency [55]. Proliferation level in cells was evaluated, as reported previously [56] using the Cell Titer96 non-radioactive cell proliferation assay kit (Promega) according to manufacturer’s protocol

### Cell invasion assay

Cell invasion were determined using 24-well microchemotaxis chambers precoated with 7.5 μg collagen type IV (Becton Dickinson Labware), as previously described [57]. Tumor cells were resuspended in serum-free media and loaded into upper chamber of each well. Cells were incubated at 37°C for 24 h in the presence or the absence of B1f and B3 compounds, after which, the filters were fixed and stained with Diff-Quik (Medion Diagnostic). Cell invasion were quantified by determination of the number of cells that migrated directly through the membrane toward the medium containing 10% serum that was used as a chemoattractant. Cells detected in each well were counted and the results were represented as (number of migrated cells/number of total cells) × 100%.

### Statistical analysis

All data are presented as ± standard error of mean (SE) unless specifically mentioned. Student’s *t* test was applied for statistical analysis, as appropriate. *P* values of <0.05 were considered significant.
